# Measurement invariance of the kidney disease and quality of life instrument (KDQOL-SF) across Veterans and non-Veterans

**DOI:** 10.1186/1477-7525-8-120

**Published:** 2010-10-25

**Authors:** Karen L Saban, Fred B Bryant, Domenic J Reda, Kevin T Stroupe, Denise M Hynes

**Affiliations:** 1 Center for Management of Chronic Complex Care, Edward Hines Jr. VA Hospital, Hines, IL, USA; 2 Loyola University Chicago, Marcella Niehoff School of Nursing, Maywood, IL, USA; 3 Loyola University Chicago, Department of Psychology, Chicago, IL, USA; 4 Veterans Affairs Cooperative Studies Program Coordinating Center, Hines, IL, USA; 5 Veterans Affairs Information Resource Center, Hines, IL, USA; 6 Loyola University Stritch School of Medicine, Maywood, IL, USA; 7 University of Illinois at Chicago, College of Medicine, Chicago, IL, USA

## Abstract

**Background:**

Studies have demonstrated that perceived health-related quality of life (HRQOL) of patients receiving hemodialysis is significantly impaired. Since HRQOL outcome data are often used to compare groups to determine health care effectiveness it is imperative that measures of HRQOL are valid. However, valid HRQOL comparisons between groups can only be made if instrument invariance is demonstrated. The Kidney Disease Quality of Life-Short Form (KDQOL-SF) is a widely used HRQOL measure for patients with chronic kidney disease (CKD) however, it has not been validated in the Veteran population. Therefore, the purpose of this study was to examine the measurement invariance of the KDQOL-SF across Veterans and non-Veterans with CKD.

**Methods:**

Data for this study were from two large prospective observational studies of patients receiving hemodialysis: 1) Veteran End-Stage Renal Disease Study (VETERAN) (N = 314) and 2) Dialysis Outcomes and Practice Patterns Study (DOPPS) (N = 3,300). Health-related quality of life was measured with the KDQOL-SF, which consists of the SF-36 and the Kidney Disease Component Summary (KDCS). Single-group confirmatory factor analysis was used to evaluate the goodness-of-fit of the hypothesized measurement model for responses to the subscales of the KDCS and SF-36 instruments when analyzed together; and given acceptable goodness-of-fit in each group, multigroup CFA was used to compare the structure of this factor model in the two samples. Pattern of factor loadings (configural invariance), the magnitude of factor loadings (metric invariance), and the magnitude of item intercepts (scalar invariance) were assessed as well as the degree to which factors have the same variances, covariances, and means across groups (structural invariance).

**Results:**

CFA demonstrated that the hypothesized two-factor model (KDCS and SF-36) fit the data of both the Veteran and DOPPS samples well, supporting configural invariance. Multigroup CFA results concerning metric and scalar invariance suggested partial strict invariance for the SF-36, but only weak invariance for the KDCS. Structural invariance was not supported.

**Conclusions:**

Results suggest that Veterans may interpret the KDQOL-SF differently than non-Veterans. Further evaluation of measurement invariance of the KDQOL-SF between Veterans and non-Veterans is needed using large, randomly selected samples before comparisons between these two groups using the KDQOL-SF can be done reliably.

## Background

The prevalence of chronic kidney disease (CKD) continues to grow each year with the incidence of patients receiving hemodialysis in the United States reaching 310 per million in 2004 [1]. Hemodialysis, while not a cure for CKD, helps prolong and improve patients' quality of life [2]. However, hemodialysis is often a burden for patients requiring them to be essentially immobile while they are connected to a dialysis machine several hours a day at least three times a week. Social activities, physical functioning and mental health are impacted due to the constraints of hemodialysis as well as from the effects of the treatment itself which can include fatigue and nausea. A number of studies have demonstrated that perceived health-related quality of life (HRQOL) of patients receiving hemodialysis is significantly impaired [3-6]. Furthermore, HRQOL has been shown to be as predictive of mortality as serum albumin levels with the latter known as being one of the strongest predictors of dialysis patient mortality[7].

Since HRQOL outcome data are often used to compare groups to determine health care effectiveness, including medication and treatment procedural effects as well as resource allocation and policy development, it is imperative that HRQOL instruments measure the same latent traits across groups. However, valid HRQOL comparisons between groups can be made only if instrument invariance is demonstrated [8]. In other words, measurement differences in HRQOL between groups should reflect true mean differences in perceived HRQOL. If group differences reflect variation in related "auxiliary" secondary dimensions of HRQOL, then the instrument is still considered to be "fair" and to reflect meaningful group differences. But if such group differences instead reflect variation in secondary dimensions that are irrelevant to HRQOL (i.e., "nuisance" factors), then the instrument is considered to reflect unfair measurement bias [9-11].

Recently, group differences in how to interpret HRQOL measures have been discussed as an issue potentially affecting the validity of comparisons between genders and different cultural groups [12-17]. For example, Mora et al., [12] found a lack of support for strict measurement invariance across African American and Latino HRQOL measures and recommended that the instrument be refined to ensure equivalence of measures across ethnic groups. In a study evaluating measurement invariance of the WHOQOL-BREF across several nations, Theuns et al.,[14] identified a significant lack of measurement invariance and cautioned researchers against using the instrument to make cross-national and cross-cultural comparisons. However, group differences are not in themselves problematic-instead, what is problematic is if these group differences do not reflect valid differences in the construct(s) being assessed. Mean differences should reflect actual group differences in the underlying attribute and should not reflect a different functioning of the measures across the different groups.

Previous studies have demonstrated that Veterans report lower HRQOL than non-Veterans with similar ages and diagnoses [18,19]. Kazis et al. [19] suggested that one possible explanation for the differences in reported HRQOL is that Veterans may experience greater psychological distress than non-Veterans. However, it must also be considered that Veterans are a cultural group with unique life experiences related to their military experience [20]. Keynan Hobbs an advanced practice psychiatric nurse and former combat Veteran eloquently describes the culture of being a Veteran, in *Reflections on the Culture of Veterans *[20]:

"More than enough evidence, from Veterans of every war, has established that combat is only the beginning of the journey. Soldiers come home, just days out of combat, and enter the purgatory that is being a Veteran. No longer true civilians, ex-soldiers enter the culture of veterans. Millions of members strong, Veterans have their own language, symbols, and gathering places where they talk about what Veterans talk about. Civilians are welcome, but it becomes apparent that they do not fit - they ask the wrong questions and say things that veterans leave unsaid. This is the way of cultures and those who belong to them." (p. 337).

The culture of Veterans may influence how Veterans interpret HRQOL measures similar to the differences in interpretation of HRQOL items found among other cultures and ethnic groups [12]. Identification of differences in HRQOL outcomes between Veterans and non-Veterans receiving hemodialysis is important for several reasons. First, HRQOL has been found to be significantly lower for patients with CKD than for the general population [21]. Thus, measuring HRQOL in CKD patients in order to measure the effectiveness of interventions to improve the lives of CKD is imperative. Second, HRQOL is a predictor of future health problems and mortality in patients (both Veterans and non-Veterans) with CKD and may help clinicians identify high risk patients in order to provide early intervention. Third, Veterans may be at a particular high risk for developing poor HRQOL because of their life experiences, socioeconomic status, etc. Valid measurement of HRQOL in Veterans is necessary to accurately assess their needs. However, a valid assessment of HRQOL in Veterans requires that the measure is functioning in a comparable manner for Veterans as it is functioning for non-Veterans. Therefore, it is imperative that HRQOL instruments be validated in Veterans prior to using to make comparisons with non-Veterans. However, prior to comparing HRQOL of Veterans with non-Veterans, it is necessary to consider measurement invariance of the instrument used to measure HRQOL. The Kidney Disease Quality of Life-Short Form (KDQOL-SF) [22] is a widely used HRQOL measure for patients with CKD, however it has not been validated in the Veteran population. Therefore, the purpose of this study was to examine the measurement invariance of the KDQOL-SF [22] instrument across Veterans and non-Veterans with CKD receiving hemodialysis. To achieve our objective, we first determined if the same factors and loadings were appropriate for both the Veteran and non-Veteran samples. We then evaluated whether the measurement structure of the KDQOL-SF was invariant across a Veteran and non-Veteran sample.

### Testing Measurement Invariance

The issue of measurement invariance concerns the degree to which the items that comprise a measurement instrument have the same meaning and measure the same constructs in the same ways across different groups of respondents. Although scores on measurement instruments are often used to compare levels of responses across different groups, such analyses of mean differences assume that the scores being contrasted are in fact comparable across groups. In this regard, several types of measurement invariance (or construct comparability) are relevant and are most often evaluated using confirmatory factor analysis (CFA) in a sequence of progressively more restrictive hypotheses about equality across groups concerning the pattern of factor loadings (configural invariance), the magnitude of factor loadings (metric invariance), and the magnitude of item intercepts (scalar invariance). In assessing factorial differences across groups, it is also important to address issues of structural invariance, or the degree to which factors have the same variances, covariances, and means across groups [23]. Although measurement invariance is a requirement for valid comparisons of group means, structural invariance is a desirable, though unnecessary precondition for meaningful group comparisons [24].

#### Partial versus total invariance

Varying degrees of measurement and structural invariance are possible across groups with respect to any or all of the invariance hypotheses, ranging from the complete absence of invariance to total invariance. Partial invariance exists when some but not all of the parameters being compared are equivalent across groups [25]. Either full or partial measurement invariance is necessary in order to permit interpretable comparisons of factor means across groups.

#### Configural invariance

An initial omnibus test of measurement invariance often entails a comparison of the covariance matrix of item variances and covariances between groups. However, numerous statistical analysts [24,26] have recommended against this overall test of equality because excellent multigroup fit in one part of the measurement model may mask departures from invariance in other parts of the model and produce Type II errors concerning overall group differences.

For this reason, focused tests of invariance typically begin by assessing the issue of equal factorial form or *configural invariance*-that is, whether the same factors and patterns of loadings are appropriate for both groups [23,27]. Configural invariance is assessed by determining whether the same congeneric measurement model provides a reasonable goodness-of-fit to each group's data [28]. Thus, whereas the tests of other forms of invariance are based on estimated *p *values associated with inferential null-hypothesis testing, the test of configural invariance is merely descriptive.

#### Metric invariance

Given configural invariance, more rigorous tests are conducted concerning first the hypothesis of equal factor loadings across groups, or *metric invariance *[23,27,29]. Also known as weak invariance [27], the issue here concerns the degree to which a one-unit change in the underlying factor is associated with a comparable change in measurement units for the same given item in each group. Items that have different factor loadings across groups represent instances of "non-uniform" differential item functioning [30,31]. Numerous theorists [23,32,33] have argued that between-group equivalence in the magnitude of factor loadings is necessary in order to conclude that the underlying constructs have the same meaning across groups.

#### Scalar invariance

Given some degree of metric invariance, a third form of measurement equivalence concerns *scalar invariance*, or the degree to which the items have the same predicted values across groups when the underlying factor mean is zero [23,27,29]. Differences in item intercepts when holding the latent variable mean constant at zero reflect instances of "uniform" differential item functioning [30,31,34,35], and indicate that the particular items yield different mean responses for individuals from different groups who have the same value on the underlying factor. Scalar invariance is tested only for items that show metric invariance [26]. Strong invariance is said to exist when equivalent form (configural invariance), equivalent loadings (metric invariance), and equivalent item intercepts (scalar invariance) are all found across groups [27].

#### Equivalence of factor variances and covariances

An additional test of structural invariance concerns the degree to which the underlying factors have the same amount of variance and covary to the same extent across groups. Although this form of invariance is unnecessary for interpretable between-group comparisons of factor means [24], the equivalence of factor variances indicates that the particular groups being compared report a comparable range of values with respect to the underlying measurement constructs; and the equivalence of factor covariances indicates that the underlying constructs interrelate to a comparable degree in each group.

#### Invariance of item unique error variances

A second test of structural invariance concerns the degree to which the underlying factors produce the same amount of unexplained variance in the items across groups. Although this form of invariance is not a technical requirement for valid between-group comparisons of factor means [32,34], the invariance of unique errors indicates that the levels of measurement error in item responses are equivalent across groups. Strict invariance is said to exist when configural invariance, metric invariance, scalar invariance, and invariance in unique errors are all found across groups [27].

#### Equivalence of factor means

A final test of structural invariance concerns whether the multiple groups have equivalent means on each underlying factor in the measurement model. The primary advantages of using CFA to compare latent variable means across groups, as opposed to comparing group means on composite indices of unit-weighted summary scores via *t *tests or ANOVAs, are that CFA allows researchers to: (a) operationalize constructs in ways that are appropriately invariant or noninvariant across groups; (b) correct mean levels of constructs for attenuation due to item unreliability; and (c) adjust between-group mean differences for differential item reliability across groups.

## Methods

### Study Design

Data for this study were from two large prospective observational studies of patients in the United States (U.S.) receiving hemodialysis: 1) Veteran End-Stage Renal Disease Study (VETERAN) sample [36] and 2) Dialysis Outcomes and Practice Patterns Study (DOPPS) sample [37,38].

#### VETERAN Sample

The VETERAN sample consisted of baseline data of 314 males between the ages of 28-85 years from a large prospective observational study of Veterans dialyzing at Department of Veterans Affairs (VA) facilities or in the private sector during 2001-2003 [36]. Veterans who had received care at a VA facility within the prior 3 years and were receiving hemodialysis for end-stage renal disease were eligible for enrollment. Patients were excluded if they: 1) had a live kidney donor identified; 2) required skilled nursing facility care; 3) had a life expectancy less than 1 year, determined by a nephrologist; 4) were cognitively impaired; 5) had a severe speech or hearing impairment; 6) were not fluent in English; or 7) had no access to a telephone for follow-up contact.

Participants were recruited from eight VA Medical Centers with outpatient dialysis facilities from 2001 to 2003 and followed for at least six months. Health-related quality of life questionnaires were completed via a phone interview. Institutional review board (IRB) approval was obtained from all VA sites. Coordinators at each site explained the study and obtained written informed consent from patients who were interested in participating.

#### Non-Veteran Sample

The non-Veteran data are from the first phase of the Dialysis Outcomes and Practice Patterns Study (DOPPS) [37,38]. The DOPPS is an international, prospective, observational study of the care and outcomes of patients receiving hemodialysis in seven countries including France, Germany, Italy, Japan, Spain, the United Kingdom, and the U.S. A detailed description of DOPPS Phase 1 has been published [37,38]. Health-related quality of life data was collected by a written questionnaire. In the U.S., 6,609 patients from 142 dialysis facilities completed baseline data between 1996 and 2001. For the present analyses, only males living in the U.S. between the ages of 28 and 85 who had completed quality of life data were included resulting in a sample size of 3,300.

Table 1 describes the demographics of the two samples.

**Table 1:** Demographics of VETERAN and DOPPS Samples

VARIABLE	VETERAN N = 314	DOPPS N = 3300
Age		
Mean years	62.14	59.68
Range	28-85 years	28-85 years
(Standard deviation)	(11.24)	(14.38)

Marital status		
Married	154 (49.36%)	1965 (61.21%)
Single	37 (11.85%)	600 (18.70%)
Divorced/Separated	86 (27.56%)	419 (13.05%)
Widowed	35 (11.22%)	226 (7.04%)

Race		
White	153 (49.35%)	1965 (59.5%)
Black	150 (48.39%)	1071 (32.5%)
Other	7 (2.26%)	260 (7.9%)

Education		
Less than high school	59 (18.91%)	426 (15.91%)
Completed high school/trade school	72 (23.08%)	514 (19.19%)
Some college	139 (44.55%)	861 (32.15%)
Completed college	35 (11.22%)	596 (22.25%)
Graduate work	7 (2.24%)	281 (10.49%)

Employed	26 (8.28%)	357 (10.81%)

Annual income		
$0 to $10,000	75 (23.89%)	716 (21.71%)
$10,000 to $20,000	100 (31.85%)	642 (19.45%)
$20,000 to $30,000	64 (20.38%)	635 (19.24%)
> $30,000	64 (20.38%)	778 (23.57%)
Not reported	11 (3.50%)	529 (16.03%)

Years since beginning dialysis	2.50 ± 2.85	2.08 ± 3.47

#### Instruments

Demographic information such as patient age, gender, marital status, race, work status, and educational level were collected using an investigator developed questionnaire.

#### Kidney Disease Quality of Life

**Figure 1:**
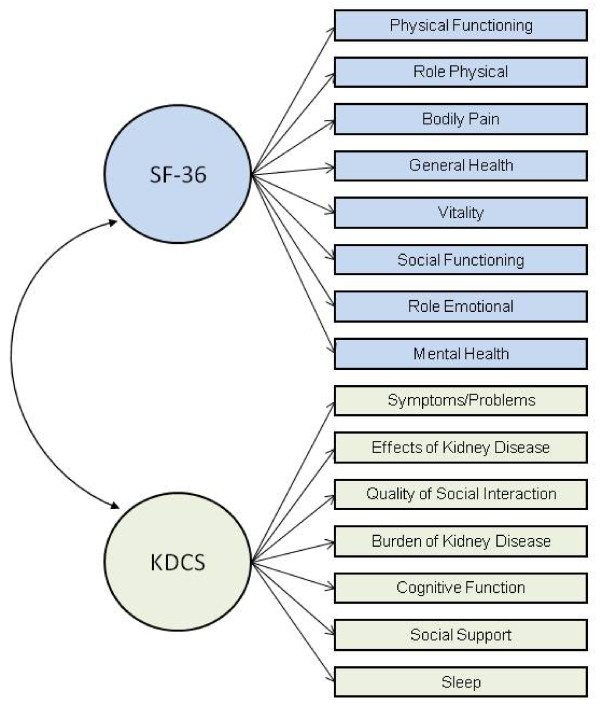
**Subscales of KDQOL**. The ellipses represent latent factors (i.e., the SF-36 and KDCS instruments), the rectangles represent measured indicators (i.e., the subscales for each instrument), the lines connecting instruments to subscales are factor loadings, and the curve connecting the two instruments represents a factor correlation. Four KDCS subscales (sexual function, work status, patient satisfaction, and staff encouragement) were not included in the confirmatory factor analysis models for this study). Because of large amounts of missing data from both the VETERANS and DOPPs samples for the sexual function subscale, sexual function was not included in the calculation of the KDCS for this study. In addition, a one-factor confirmatory factor analysis of the KDCS demonstrated weak factor loadings of the subscales of work status, patient satisfaction and dialysis staff encouragement suggesting that these three subscales measure something other than HRQOL. Therefore, these four subscales were not included in our measurement models (see data analysis section for further details).

Health-related quality of life was measured with the Kidney Disease Quality of Life Instrument -Short Form (KDQOL-SF). The KDQOL was developed as a self-report, health-related quality of life measurement tool designed specifically for patients with CKD [22]. The 134-item KDQOL was later condensed into the 80-item Kidney Disease Quality of Life Instrument-Short Form (KDQOL-SF) [39]. The questionnaire consists of the generic SF-36 [40] as well as 11 multi-item scales focused on quality of life issues specific to patients with kidney disease (Figure 1). Subscales of the KDCS are (1) symptoms/problems (6 items), (2) effects of kidney disease (4 items), (3) burden of kidney disease (3 items), (4) work status (2 items), (5) cognitive function (3 items), (6) quality of social interaction (3 items), (7) sexual function (2 items), (8) sleep (4 items), (9) social support (2 items), (10) dialysis staff encouragement (2 items), and (11) patient satisfaction. For example, related to the effects of kidney disease, participants are asked how true or false (using a 5 point Likert scale ranging from "definitely true" to "definitely false" the following statements are for them: (1) "My kidney disease interferes too much with my life;" and (2) "Too much of my time is spent dealing with my kidney disease" [22,39]. All kidney disease subscales are scored on a 0 to 100 scale, with higher numbers representing better HRQOL. The 11 kidney disease-specific subscales can be averaged to form the Kidney Disease Component Summary (KDCS) [21,41-44]. The KDQOL-SF has been widely used in several studies of patients with kidney disease, including the ongoing, international DOPPS [21,45-50], and has demonstrated good test-retest reliability on most dimensions [2,22]. Published reliability statistics for all subscales range from 0.68 to 0.94 with the subscale of social interaction (0.68) being the only subscale with an internal consistency reliability of less than the recommended 0.70 [22].

#### Data Analysis

Missing values occurred between 1% and 10% for all items except for sexual function which was missing greater than 50% of data. The Veteran data set contained less missing data than the DOPPS data set (between 0 to 5% for the Veterans versus 6% to 10% missing data for the DOPPS data set). This difference may have been attributed to the Veteran data being collected over the telephone whereas DOPPS data were collected via written questionnaire. Because of the large amounts of missing data from both the VETERANS and DOPPs samples for the sexual function subscale, sexual function was not included in the calculation of the KDCS. For all other items, missing data were replaced for the KDQOL-SF variables using the SAS 9.2 (Cary, NC) multiple imputation procedure [51]. The multiple imputation procedure consisted of using a regression model fitted for each variable with missing data with 3 imputed data sets [52]. A one-factor confirmatory factor analysis of the KDCS demonstrated weak factor loadings of the subscales of work status, patient satisfaction and dialysis staff encouragement, suggesting that these three subscales measure something other than HRQOL. These findings are consistent with CFA findings from a previous study [53]. Therefore, the 7-subcale KDCS comprising the subscales measuring symptoms, effects of kidney disease on daily life, quality of social interaction, burden of kidney disease, cognitive function, support, and sleep was used for analyses in this study. Descriptive statistics (mean, range, standard deviation) were calculated using SAS (Cary, NC).

### Analytic Strategy

CFA. We used single-group confirmatory factor analysis (CFA) via LISREL 8 [28] to evaluate the goodness-of-fit of the hypothesized measurement model for responses to the subscales of the KDCS and SF-36 instruments when analyzed together; and given acceptable goodness-of-fit in each group, we then used multigroup CFA to compare the structure of this factor model in the VETERAN (*N *= 314) and DOPPS (*N *= 3,300) samples. As a first step, we evaluated separately for each group the goodness-of-fit of a CFA model that specified two correlated factors consisting of the seven subscales of the KDCS and the eight subscales of the SF-36. The rationale for examining a two-factor, second-order structure considering generic HRQOL as one factor and disease-specific HRQOL as another factor is supported by the literature in which generic HRQOL and disease-specific HRQOL are considered to be distinct, yet complementary concepts [54]. In a seminal review, Patrick and Deyo [54] describe an approach to measuring HRQOL using both a generic instrument and condition disease-specific measure with the intention "not to measure the same concepts as a generic measure with specific reference to a medical condition, but to capture the additional, specific concerns of patient with the condition that are not contained in generic measures" (p. S224). Furthermore, several studies have found evidence that generic and disease-specific HRQOL instruments measure discrete concepts. For example, Bombardier et al., in a comparison of a generic (SF-36) and a disease-specific HRQOL measure (Western Ontario and McMaster Universities Osteoarthritis Index) in patients after knee surgery found that the disease-specific measure detected improvements post-surgery whereas the SF-36 discriminated better among participants' pain and functional level [55]. Other studies have also found that generic and disease-specific HRQOL measure different aspect of HRQOL concluding that both types of instruments should be included in studies [56-58].

CFA models were analyzed via maximum-likelihood estimation using the covariance matrix of the KDCS and SF-36 subscales. Because HRQOL responses tend to be distributed nonnormally and because nonnormality inflates the goodness-of-fit chi-square, reduces standard errors, and exaggerates statistical significance, we also analyzed the KDCS and SF-36 data using robust maximum likelihood estimation, by analyzing the asymptotic covariance matrices to estimate the Satorra-Bentler scaled chi-square value [59]. An identical pattern of results emerged as when using traditional maximum-likelihood estimation, although the goodness-of-fit chi-square values were generally smaller. For present purposes, we have chosen to report results using traditional maximum-likelihood estimation.

To define the units of variance for each factor in single-group CFA, we standardized the KDCS and SF-36 factors by fixing their variances at 1.0. To define the units of variance for the factors in the multigroup CFA models, we identified a single subscale for each factor that had a virtually identical loading for both groups and then fixed this loading to a value of 1.0 for each group [28,60]. For the KDCS factor, we selected the Symptoms subscale as the referent item because it had practically the same loading for both groups in the completely standardized single-group CFA solutions: 0.750 for Veteran sample and 0.747 for the DOPPS sample. And for the SF-36 factor, we selected the Role Physical (RP) subscale as the referent item because it had practically the same loading for both groups in the completely standardized single-group CFA solutions: 0.614 for VETERAN sample and 0.611 for the DOPPS sample.

#### Assessing model fit

We used four different statistical criteria to judge the goodness-of-fit of the hypothesized two-factor CFA model. As measures of absolute fit, we examined the root mean square error of approximation (RMSEA) and the standardized root mean residual (SRMR). RMSEA reflects the size of the residuals that result when using the model to predict the data, adjusting for model complexity, with smaller values indicating better fit. According to Browne and Cudeck [61], RMSEA < .05 represents "close fit," RMSEA between .05 and .08 represents "reasonably close fit," and RMSEA > .10 represents "an unacceptable model." SRMR reflects the average standardized absolute value of the difference between the observed covariance matrix elements and the covariance matrix elements implied by the given model, with smaller values indicating better fit. Hu and Bentler [62] suggested that SRMR < .08 represents acceptable model fit. As measures of relative fit, we used the non-normed fit index (NNFI) and the comparative fit index (CFI). NNFI and CFI indicate how much better the given model fits the data relative to a "null" model that assumes sampling error alone explains the covariation observed among items (i.e., no common variance exists among measured variables). Bentler and Bonett [63] recommended that measurement models have NNFI and CFI > .90. More recently, Hu and Bentler [64] suggested that relative fit indices above 0.95 indicate acceptable model. However, Marsh et al., [65] have strongly cautioned researchers against accepting Hu and Bentler's (1999) [64] more stringent criterion for goodness-of-fit indices, and have provided a strong conceptual and statistical rationale for retaining Bentler and Bonett's [63] long-standing criterion for judging the acceptability of goodness-of-fit indices. Therefore, following Marsh et al.'s [65] recommendation, we have adopted Bentler and Bonett's [63] criterion of relative fit indices > .90 as reflective of acceptable model fit.

#### Assessing invariance

We followed Vandenberg and Lance's [23] recommended sequence for conducting tests of measurement invariance. Given an acceptable fit for the hypothesized two-factor CFA model in each group (i.e., configural invariance), we tested five different hypotheses about measurement invariance between the VETERAN and DOPPS samples. These structural hypotheses concerned between-group differences (versus equivalence) in: (a) the magnitude of the factor loadings (metric invariance); (b) the intercepts of the measured subscales (scalar invariance); (c) the variances and covariance of the KDCS and SF-36 factors; (d) the unique error variances of the measured subscales; and (e) the latent means of the KDCS and SF-36Q factors.

We used the difference in chi-square values and degrees of freedom, i.e., the likelihood ratio test [66], to test hypotheses about differences in goodness-of-fit between nested CFA models. Because the goodness-of-fit chi-square is inflated by large sample size [66], we also examined differences in CFI across nested models, with difference in the CFI (ΔCFI) ≤ .01 considered evidence of measurement invariance [67]. In addition, we computed the effect size for each probability-based test of invariance expressed in terms of *w*^2^, or the ratio of chi-square divided by *N *[68], which is analogous to *R*-squared (i.e., the proportion of explained variance) in multiple regression. Cohen [68] suggested that *w*^2 ^≤ 0.01 is small, *w*^2 ^= 0.09 is medium, and *w*^2 ^≥ 0.25 is large.

In testing invariance hypotheses, there is disagreement in the literature about whether researchers should test invariance hypotheses globally across all relevant parameters simultaneously (e.g., a single test of whether all factor loadings show between-group invariance) versus test invariance hypotheses separately across relevant sets of parameters (e.g., separate tests of the equivalence of factor loadings for each factor). Although omnibus tests of parameter equivalence reduce Type I errors by decreasing the number of statistical tests when the null hypothesis is true, Bontempo and Hofer [24] have suggested that perfectly invariant factors can obscure noninvariant factors and make multivariate global tests of invariance misleading. For this reason, we chose to examine the between-group equivalence of factor loadings, item intercepts, and unique error variances separately for each factor in our two-factor CFA model.

To further reduce the likelihood of capitalizing on chance, we corrected the Type I error rate for probability-based tests of invariance (see Cribbie) [69], by imposing a sequentially-rejective Bonferroni adjustment to the generalized *p *value for each statistical test [70]. Specifically, we used a Sidak step-down adjustment procedure [71,72] to ensure an experimentwise Type I error rate of *p *< .05, correcting for the number of statistical comparisons made.

In drawing inferences from tests of measurement or structural invariance, we examined four different statistical criteria: (a) the unadjusted *p*-value associated with the likelihood-ratio test; (b) the sequentially-rejective Bonferroni adjusted *p*-value associated with the likelihood-ratio test; (c) the difference in CFI values (ΔCFI); and (d) effect size (*w*^2^). Research comparing the likelihood-ratio test and ΔCFI as criteria for judging measurement invariance [73] suggests that these two criteria produce highly inconsistent conclusions. Because the likelihood-ratio test is biased against finding invariance when sample sizes are large [67,73,74], we expected that likelihood-ratio tests using unadjusted *p*-values would more often support the rejection of invariance hypotheses relative to the other statistical criteria, given the large sample size for our multigroup analyses (*N *= 3,614). Because the large number of anticipated invariance tests (i.e., 40-50) will produce a more stringent adjusted *p*-value, we expected that using Bonferroni-adjusted *p *values would reduce the bias toward rejecting invariance hypotheses via the likelihood-ratio test.

## Results

### Single-Group CFA Modeling

#### Configural invariance

CFAs revealed that the hypothesized two-factor model fit the data of both the VETERAN and DOPPS samples reasonably well, *χ*^2^(89, *N *= 314) = 331.632, RMSEA = .091, SRMR = .058, NNFI = .952, CFI = .959, and *χ*^2^(89, *N *= 3,300) = 2464.593, RMSEA = .086, SRMR = .051, NNFI = .956, CFI = .963, respectively. Table 2 presents the within-group completely standardized CFA solutions (in which factor variances and item variances were both fixed at 1.0) for the VETERAN and DOPPS samples. These results establish the configural invariance of the two-factor measurement model, whereby the same two factors (KDCS and SF-36) and the same pattern of factor loadings are relevant for both the VETERAN and DOPPS samples. Further supporting the configural invariance of the hypothesized two-factor model, squared multiple correlations (i.e., proportions of variance explained by the relevant factor) for the subscales reflecting each factor were generally large for each factor in both groups: VETERAN sample, KDCS median *R*^2 ^= .394, SF-36 median *R*^2 ^= .484; DOPPS sample, KDCS median *R*^2 ^= .398, SF-36 median *R*^2 ^= .459.

**Table 2:** Within-Group Completely Standardized Factor Loadings and Squared Multiple Correlations for VETERAN (*N *= 314) and DOPPS (*N *= 3,300) Samples for the Two-Factor CFA Model

Subscales	Factors				Squared Multiple Correlations	
			
	KDCS		SF-36			
	
	VETERAN	DOPPS	VETERAN	DOPPS	VETERAN	DOPPS
Burden of Kidney Disease	.593	.697	- -	- -	.351	.485

Quality of Social Interaction	.628	.591	- -	- -	.394	.349

Cognitive Functioning	.655	.631	- -	- -	.429	.398

Symptoms/Problems	.750	.747	- -	- -	.562	.558

Effects of Kidney Disease	.728	.733	- -	- -	.530	.537

Sleep	.618	.584	- -	- -	.382	.341

Social Support	.516	.392	- -	- -	.266	.154

PF	- -	- -	.523	.580	.273	.336

RP	- -	- -	.614	.611	.377	.373

BP	- -	- -	.714	.678	.510	.459

GH	- -	- -	.676	.725	.457	.526

MH	- -	- -	.743	.713	.551	.508

RE	- -	- -	.564	.586	.318	.344

SF	- -	- -	.761	.784	.579	.614

VT	- -	- -	.718	.763	.516	.583

*Note*. CFA = confirmatory factor analysis. Completely standardized factor loadings are regression coefficients obtained in predicting subscale scores when factors and subscales are both standardized. Squared multiple correlations represent the proportion of variance in each subscale that the underlying factor explains. Blank loadings were fixed at zero in the CFA model. PF = Physical Functioning. RP = Role Physical. BP = Bodily Pain. GH = General Health. MH = Mental Health. RE = Role Emotional. SF = Social Functioning. VT = Vitality.

In the completely standardized CFA solution, the KDCS and SF-36 factors correlated 0.924 in the VETERAN sample and 0.879 in the DOPPS sample. Although these factor intercorrelations reflect a high degree of overlap between the two HRQOL instruments in both the VETERAN (0.924^2 ^= 85% shared variance) and DOPPS (0.879^2 ^= 77% shared variance) samples, they also indicate that roughly one-seventh of the variance in each instrument for the VETERAN sample, and one-quarter of the variance in each instrument for the DOPPS sample, has nothing to do with the other instrument. Furthermore, a one-factor model, representing overall HRQOL, fit the combined KDCS and SF-36 data significantly worse than did the two-factor model for both the VETERAN, Δ*χ*^2^(1, *N *= 314) = 20.287, *p *< .0001, and DOPPS samples, Δ*χ*^2^(1, *N *= 314) = 524.571, *p *< .0001; and a one-factor model did not yield an acceptable model fit with respect to RMSEA for either the VETERAN, *χ*^2^(90, *N *= 314) = 352.459, RMSEA = .102, SRMR = .0589, NNFI = .946, CFI = .954, or DOPPS sample, *χ*^2^(90, *N *= 3,300) = 2989.164, RMSEA = .107, SRMR = .0564, NNFI = .942, CFI = .951.

Although the two-factor model fit the data well, we also tested a three-factor model that consisted of a single second-order factor for the KDCS and two second-order factors, representing the physical and mental component summary scores of the SF-36 [40]. The two second-order factors were evaluated by allowing the four physical subscales (PF, RP, BP, & GH) to load on the second-order physical component summary and the four mental health subscales (MH, RE-SF, & VT) to load on the second-order mental health component summary and then estimating these loadings. This three-factor model fit the data of both the DOPPS and VETERAN sample slightly better than the two-factor model. However, the SF-36 physical component summary factor correlated very highly with the SF-36 mental component summary in the CFA solution for both the DOPPS sample (r=.957) and the VETERAN sample (r=.997).

### Multigroup CFA Modeling

Having established configural invariance (or an identical pattern of factor loadings), we next used multigroup CFA to assess a set of increasingly restrictive hypotheses concerning measurement invariance across the two samples. Analyzing the data for the VETERAN and DOPPS samples in a multigroup model with no cross-group invariance constraints provided the baseline model for subsequent tests of invariance, (see Model 1, Table 3).

**Table 3:** Results of tests of invariance for the VETERAN (*N *= 314) and DOPPS (*N *= 3,300) samples

			Comparative Statistics						
			
Model	***χ***^**2**^	*df*	Contrast with Model #	**Δ*χ***^**2**^	Δ*df*	Unadj. *p *<	Bonf. Adj. *p *<	ΔCFI	***w***^**2**^
1. Baseline model: Two factors (KDCS & SF-36) with no invariance constraints	2796.225	178	- -	- -	- -	- -	- -	- -	- -

2. KDCS factor loadings invariant	2819.092	184	1	22.867	6	.00085	.025	.0003	.08

3. SF-36 factor loadings invariant	2804.771	185	1	8.546	7	.29	ns	.0002	.05

4. KDCS Burden subscale loading invariant	2796.239	179	1	0.014	1	.91	ns	<.0001	<.01

5. KDCS Social Interaction subscale loading invariant	2799.730	179	1	3.505	1	.062	ns	.0004	.03

6. KDCS Cognitive subscale loading invariant	2796.928	179	1	0.703	1	.41	ns	<.0001	.01

7. KDCS Effects subscale loading invariant	2798.687	179	1	2.462	1	.12	ns	.0005	.03

8. KDCS Sleep subscale loading invariant	2811.091	179	1	14.866	1	.00012	.0036	.0003	.06

9. KDCS Social Support subscale loading invariant	2803.528	179	1	7.303	1	.0069	ns	.0001	.04

10. Partially metric invariant model (factor loadings for KDCS Sleep & Social Support subscales noninvariant)	2810.567	189	1	14.342	11	.22	ns	.0003	.06

11. Partially invariant model with 5 metric invariant KDCS subscale intercepts invariant	2894.471	194	10	83.904	5	.000001	.00005	.0019	.15

12. Partially invariant model with 8 metric invariant SF36 subscale intercepts invariant	2964.251	197	10	153.684	8	.000001	.00005	.0040	.21

13. Partially invariant model with intercept of KDCS Burden subscale invariant	2812.836	190	10	2.269	1	.14	ns	.0003	.03

14. Partially invariant model with intercept of KDCS Social Interaction subscale invariant	2838.461	190	10	27.894	1	.000001	.00005	.0008	.09

15. Partially invariant model with intercept of KDCS Cognitive subscale invariant	2835.202	190	10	24.635	1	.000001	.00005	.0007	.08

16. Partially invariant model with intercept of KDCS Symptoms subscale invariant	2877.711	190	10	67.144	1	.000001	.00005	.0015	.14

17. Partially invariant model with intercept of KDCS Effects subscale invariant	2839.951	190	10	29.384	1	.000001	.00005	.0008	.09

18. Partially invariant model with intercept of SF-36 PF subscale invariant	2815.734	190	10	5.167	1	.024	ns	.0004	.04

19. Partially invariant model with intercept of SF-36 RP subscale invariant	2846.345	190	10	35.778	1	.000001	.00005	.0001	.10

20. Partially invariant model with intercept of SF-36 BP subscale invariant	2819.639	190	10	9.072	1	.0026	ns	.0004	.05

21. Partially invariant model with intercept of SF-36 GH subscale invariant	2810.568	190	10	0.001	1	.98	ns	.0003	<.01

22. Partially invariant model with intercept of SF-36 MH subscale invariant	2837.769	190	10	27.202	1	.000001	.00005	.0008	.09

23. Partially invariant model with intercept of SF-36 RE subscale invariant	2900.352	190	10	89.785	1	.000001	.00005	.0018	.16

24. Partially invariant model with intercept of SF-36 SF subscale invariant	2831.587	190	10	21.020	1	.000005	.00016	.0007	.08

25. Partially invariant model with intercept of SF-36 VT subscale invariant	2810.914	190	10	0.347	1	.56	ns	.0003	<.01

26. Partially metric invariant model with two-factor variances & covariance invariant	2816.786	192	10	6.219	3	.11	ns	.0005	.04

27. Partially metric invariant model with factor variances-covariance & unique error variances for KDCS subscales invariant	2866.086	199	26	49.300	7	.000001	.00005	.0007	.12

28. Partially metric invariant model with factor variances-covariance & unique error variances for SF-36 subscales invariant	2840.570	200	26	23.784	8	.0025	ns	<.0001	.09

29. Partially metric invariant model with factor variances-covariance & unique error variance for KDCS Burden subscale invariant	2827.202	193	26	10.416	1	.0013	.036	.0003	.07

30. Partially metric invariant model with factor variances-covariance & unique error variance for KDCS Social Interaction subscale invariant	2816.909	193	26	0.123	1	.73	ns	.0006	.01

31. Partially metric invariant model with factor variances-covariance & unique error variance for KDCS Cognitive subscale invariant	2821.228	193	26	4.442	1	.036	ns	.0001	.04

32. Partially metric invariant model with factor variances-covariance & unique error variance for KDCS Symptoms subscale invariant	2825.083	193	26	8.297	1	.004	ns	.0001	.05

33. Partially metric invariant model with factor variances-covariance & unique error variance for KDCS Effects subscale invariant	2816.917	193	26	0.131	1	.72	ns	.0006	.01

34. Partially metric invariant model with factor variances-covariance & unique error variance for KDCS Sleep subscale invariant	2838.330	193	26	21.544	1	.000004	.00013	<.0001	.08

35. Partially metric invariant model with factor variances-covariance & unique error variance for KDCS Social Support subscale invariant	2821.074	193	26	4.288	1	.039	ns	.0009	.03

36. Partially metric invariant model with factor variances-covariance & unique error variance for SF-36 PF subscale invariant	2817.060	193	26	0.274	1	.61	ns	.0006	.01

37. Partially metric invariant model with factor variances-covariance & unique error variance for SF-36 RP subscale invariant	2818.194	193	26	1.408	1	.24	ns	.0004	.02

38. Partially metric invariant model with factor variances-covariance & unique error variance for SF-36 BP subscale invariant	2816.855	193	26	0.069	1	.80	ns	.0007	<.01

39. Partially metric invariant model with factor variances-covariance & unique error variance for SF-36 GH subscale invariant	2819.464	193	26	2.678	1	.11	ns	.0003	.03

40. Partially metric invariant model with factor variances-covariance & unique error variance for SF-36 MH subscale invariant	2817.791	193	26	1.005	1	.32	ns	.0009	.02

41. Partially metric invariant model with factor variances-covariance & unique error variance for SF-36 RE subscale invariant	2821.873	193	26	5.087	1	.025	ns	.0011	.09

42. Partially metric invariant model with factor variances-covariance & unique error variance for SF-36 SF subscale invariant	2821.253	193	26	4.467		.035	ns	.0002	.04

43. Partially metric invariant model with factor variances-covariance & unique error variance for SF-36 VT subscale invariant	2826.729	193	26	9.943		.0017	.045	.0002	.05

*Note: *CFI = Comparative fit index. W^2 ^= ratio of chi-square divided by *N *[68], which is analogous to *R*-squared (i.e., the proportion of explained variance) in multiple regression. Cohen [68] suggested that *w*^2 ^≤ 0.01 is small, *w*^2 ^= 0.09 is medium, and *w*^2 ^≥ 0.25 is large.

#### Metric invariance

In the next step, we examined the magnitude of factor loadings or metric invariance. As seen in Table 3 (Model 3), the likelihood-ratio test revealed invariant factor loadings for the SF-36 subscales according to both unadjusted (*p *< .29) and Bonferroni-adjusted (*p *= ns) criteria. In addition, the effect size of group differences in loadings on the SF-36 factor was modest (*w*^2 ^= .05), and the change in CFI (ΔCFI = .0002) also suggested invariant SF-36 factor loadings. In contrast, the likelihood-ratio test revealed significant group differences in loadings for the KDCS factor (Model 2) according to both unadjusted (*p *< .00085) and Bonferroni-adjusted (*p *< .025) criteria. However, this effect approached only medium size (*w*^2 ^= .08), and the change in CFI (ΔCFI = .0003) suggested that the VETERAN and DOPPS samples had equivalent loadings on the KDCS factor.

Tests of the invariance of factor loadings for each of the non-referent KDCS subscales revealed statistically significant differences in loadings for two subscales (Sleep and Social Support) using the unadjusted criterion (*p *< .0069), but for only the Sleep subscale using the adjusted criterion (*p *< .0036; see Table 3, Model 8). All six tests of invariance in KDCS subscale factor loadings produced modest effect sizes (*w*^2^s ≤ .06), and all ΔCFIs were within the recommended 0.01 threshold for inferring invariance (ΔCFIs ≤ .0005).

Adopting the most conservative criterion for assessing invariance (i.e., unadjusted *p*-value), we thus sought to establish a partially metric invariant measurement model that constrained the factor loadings for all seven non-referent SFQ subscales and four of the six non-referent KDCS subscales (all except the Sleep and Social Support subscales) to be invariant across the VETERAN and DOPPS samples. This partially metric invariant model fit the data well and provided an equivalent goodness-of-fit compared to the initial unconstrained baseline model, Δ(11, *N *= 3,614) = 14.342, unadjusted *p *< .22, Bonferroni-adjusted *p *= ns, ΔCFI = .0003, *w*^2 ^= .06 (see Model 10, Table 3). These results support the conclusion that the VETERAN and DOPPS samples used the SF-36 subscales in largely equivalent ways to define the subjective quality of their lives (full metric equivalence). Thus, quality of life, as measured by the KDCS and SF-36, has mostly the same meaning for the VETERAN and DOPPS samples (weak invariance).

#### Scalar invariance

As discussed, scalar invariance is the magnitude of item intercepts. According to the likelihood-ratio test (unadjusted *p *< .000001, Bonferroni-adjusted *p *< .00005), an omnibus test of scalar invariance suggested that the five metric-invariant KDCS subscales (including the referent subscale) had different intercepts for the VETERAN and DOPPS samples (see Table 3, Model 11). Although the size of this overall effect was between medium and large (*w*^2 ^= .15), the difference in CFI again did not reach the 0.01 threshold (ΔCFI = .0019). As seen in Table 3, individual follow-up tests of scalar invariance revealed that only the KDCS Burden subscale (Model 13) had equivalent intercepts for the two groups, as reflected in a nonsignificant likelihood-ratio test (unadjusted *p *< .14, Bonferroni-adjusted *p *= ns), ΔCFI <0.01, and a small effect size (*w*^2 ^= .03).

Likewise, an omnibus test of scalar invariance suggested that the eight metric-invariant SF-36 subscales (including the referent subscale) had different intercepts for the VETERAN and DOPPS samples, according to the likelihood-ratio test (unadjusted *p *< .000001, Bonferroni-adjusted *p *< .00005). Although the size of this overall effect was relatively large (*w*^2 ^= .21), the difference in CFI again did not reach the 0.01 threshold (ΔCFI = .004). As seen in Table 3 (Models 18 thru 25), individual follow-up tests of scalar invariance revealed that four of the SF-36 subscales (PF, BP, GH, & VT) had equivalent intercepts for the two groups, as reflected in nonsignificant Bonferroni-adjusted likelihood-ratio tests, ΔCFIs < 0.01, and small effect sizes (*w*^2^s ≤ .05).

#### Strong invariance

Thus, supporting partial scalar invariance (magnitude of item intercepts), half of the eight SF-36 subscales, but only one of the seven KDCS subscales, showed evidence of scalar invariance for the VETERAN and DOPPS groups. These results suggest that strong invariance (i.e., configural, metric, and scalar combined) exists in partial (50%) form for the SF-36, but only minimal form (one subscale) for the KDCS. Inspection of CFA solutions revealed that subscales with nonequivalent intercepts had higher values for the VETERAN sample relative to the DOPPS sample.

#### Invariance of factor variances and covariance

As seen in Table 3 (Model 26), all four statistical criteria suggested that the variances and covariance of the KDCS and SF-36 factors were equivalent for the VETERAN and DOPPS samples. These results indicate the two groups used an equivalent range of the latent construct continuum in responding to the subscales for each instrument [23]. Because both the factor variances and the covariance are invariant, the correlation between the factors is also invariant [26].

The finding of equal factor variances across groups is important for at least two reasons. First, the invariance of factor variances is a precondition for using the comparison of item unique variances as a test of equal subscale reliabilities across groups [23,75]. Second, because factor variances are equal across groups, regression coefficients obtained when predicting the factors from other constructs are not biased by differential range restriction across groups [24].

#### Invariance of item unique error variances

As seen in Table 3 (Model 27), the omnibus hypothesis of invariance in unique error variances for the KDCS subscales (*w*^2 ^= .12) was rejected according to the likelihood-ratio test (adjusted *p *< .000001, Bonferroni-adjusted *p *< .00005), but not according to the difference in CFI values (ΔCFI = .0007). Individual follow-up tests (*w*^2^s ≤ .05) revealed that five of the KDCS subscales-Social Interaction, Cognitive Functioning, Symptoms, Effects, and Social Support-had invariant unique error variances according to the Bonferroni-adjusted likelihood-ratio test (all *p*s = ns) and the difference in CFI (all ΔCFIs < .001). These results suggest that five (71%) of the seven KDCS subscales were equally reliable for the VETERAN and DOPPS samples. Both of the KDCS subscales (Burden and Sleep) that showed evidence of differential reliability had greater unique error variance for the VETERAN sample than for the DOPPS sample in the partially invariant CFA model.

Also seen in Table 3 (Model 28), the omnibus hypothesis of invariance in unique error variances for the SF-36 subscales (*w*^2 ^= .09) was rejected according to the unadjusted likelihood-ratio test (*p *< .0025), but not according to the Bonferroni-adjusted likelihood-ratio test (*p *= ns) or the difference in CFI (all ΔCFIs < .0001). Individual follow-up tests (*w*^2^s ≤ .05) revealed that five of the SF-36 subscales-PF, RP, BP, GH, and MH-had invariant unique error variances according to the likelihood-ratio test (all unadjusted *p*s > .11, all Bonferroni-adjusted *p*s = ns) and the difference in CFI (all ΔCFIs < .001). In addition, the RE subscale (*w*^2 ^= .09) and SF subscale (*w*^2 ^= .04) of the SF-36 had invariant unique error variances according to the Bonferroni-adjusted likelihood ratio test (*p*s = ns), but not according the unadjusted likelihood-ratio test (*p*s < .035) or the difference in CFI (ΔCFIs < .0012). And the VT subscale of the SF-36 had a different unique error variance for the two groups according to the likelihood-ratio test (unadjusted *p *< .0017, Bonferroni-adjusted *p *< .045), but had a small ΔCFI (.0002) and a small effect size (*w*^2 ^= .05). These results suggest that at least seven (88%) of the eight SF-36 subscales were equally reliable for the VETERAN and DOPPS samples. The one SF-36 subscale that showed evidence of differential reliability (VT) had greater unique error variance for the VETERAN sample than for the DOPPS sample.

#### Invariance in factor means

As a final step in our invariance analyses, we tested for group differences in latent means for the KDCS and SF-36 factors, using a multigroup CFA model that specified partially invariant factor loadings (all loadings invariant except for the Sleep and Social 2 subscales of the KDCS), fully invariant factor variances and covariances, and partially invariant unique error variances (all unique error variances invariant except for the Burden and Sleep subscales of the KDCS and the VT subscale of the SF-36). Because of problems of under-identification, item intercepts and factor means cannot both be estimated in the same model [76] nor can the factor means of both groups be estimated simultaneously [28]. Accordingly, to test invariance in factor means, we followed standard practice in the structural equation modeling literature by: (a) constraining all item intercepts to be equal across groups; (b) fixing at zero the factor means for the DOPPS group; (c) estimating the factor means for the VETERAN group; and (d) using Wald tests to assess whether the factor means for the VETERAN group were significantly different from zero (i.e., from the factor means for the DOPPS group).

This final partially invariant multigroup CFA model fit the data of the two groups reasonably well. Inspection of the LISREL solution revealed that the latent means were significantly higher for the VETERAN sample, compared to the DOPPS sample, for both the KDCS (*Z *= 5.253, *p *< .000001, Cohen's *d *= 0.18) and SF-36 (*Z *= 7.240, *p *< .000001, Cohen's *d *= 0.23) factors. Thus, given that higher scores reflect higher quality of life, the VETERAN sample reported higher overall levels of subjective life quality on both the general and specific measures of QOL, when controlling for differences in the ways in which the two groups used the subscales to define QOL and for between-group differences in the reliabilities of the subscales.

## Discussion

This study evaluated the measurement invariance of the KDQOL-SF in a sample of Veterans and non-Veterans with CKD receiving hemodialysis. Confirmatory factor analyses demonstrated that the hypothesized two-factor model (KDCS and SF-36) fit the data of both the VETERAN and DOPPS samples well, supporting configural invariance. We also tested a three-factor model using a single, second-order factor for the KDCS and two second-order factors for the SF-36 (physical and mental component summaries) and found that although this three-factor model fit the data of both the DOPPS and VETERAN samples slightly better than our two-factor model, the SF-36 physical health component correlated very highly with the mental health component. These findings strongly suggest that a single second-order factor is a more appropriate way of modeling the SF-36 in the combined data analysis than is the two second-order SF-36 factors. In fact, when we attempted to model two second-order factors for the SF-36 in the combined analysis, it resulted in an inadmissible PHI matrix of factor correlations providing more evidence that only one second-order factor better represents the data. Our finding of a single second-order factor for the SF-36 data is consistent with other published studies. For example, in a study of 339 patients with Parkinson's disease, confirmatory factor analysis demonstrated evidence to support a single second-order factor for the SF-36 data but did not find evidence to support two second-order factors for physical and mental health components [77]. Earlier studies have also found support for a one second-order factor for the SF-36 [78,79].

Although the disease-specific HROQL (KDCS) and generic HRQOL (SF-36) components of the KDQOL-SF were highly correlated with one another, a one-factor model in which the KDCS and SF-36 represented overall HRQOL provided an unacceptable model fit, whereas a two-factor model fit the data reasonably well. These findings suggest that the KDCS and SF-36 measure two similar but distinct factors. It should be noted that the KDCS and HRQOL components were allowed, but not forced to correlate with one another. Our findings are consistent with several other studies demonstrating that generic measures of HRQOL and disease-specific measures make a unique contribution to the understanding of HRQOL [54,80-82].

As more than half of the data were missing for the sexual function scale of the KDCS, we did not include this subscale in our analysis. Other studies have also reported significant amounts of missing data for the KDCS sexual functioning subscale [2]. A possible reason for this missing data may include the sensitive nature of the questions for this particular subscale. Further evaluation and possible revision of the questions included in the sexual functioning subscale may be necessary to better capture this aspect of health-related quality of life.

Multigroup CFA results concerning configural, metric, and scalar invariance suggested the partial strict invariance for the SF-36, but only weak invariance for the KDCS. Overall, these results support the conclusion that the SF-36 has more equivalent psychometric properties for the VETERAN and DOPPS samples than does the KDCS. Thus, the more general measure of HRQOL appears to provide greater measurement equivalence across samples than does the more specific measure of QOL related to kidney disease. The SF-36 has been more widely validated in different cultural groups than the more recently developed KDCS.

The VETERAN sample tended to endorse higher scores on the same rating scales than the DOPPS sample, thus reporting higher overall HRQOL levels for both the generic (SF-36) and disease-specific (KDCS) factors. This finding is contrary to results of previous studies in which Veterans tend to report lower HRQOL than non-Veterans [18,19]. We speculate that Veterans receiving dialysis, in particular those receiving dialysis at a VA dialysis center, may have a greater opportunity to socialize with others with similar backgrounds and experiences as themselves during the 12-15 hours per week of hemodialysis. Increased social support may have led to better reported HRQOL for Veterans. On the other hand, response bias must also be considered. It is possible that our Veteran subjects, who were interviewed over the phone, provided responses to the interviewer that they believed to be socially desirable. More research is needed to clarify this finding.

The issue of how to interpret regression intercept differences between groups has been widely discussed in the literature [83-85]. Millsap [83] concluded that intercept differences may reflect actual mean group differences and may not necessarily reflect measurement bias. Furthermore, Millsap [83] suggested that unreliability may contribute to group differences found in intercepts. Therefore, we interpret our findings of higher subscale intercepts for the VETERAN sample compared to the DOPPS sample as reflecting higher latent mean scores for the VETERAN sample as compared to the DOPPS sample. Consistent with Millsap's [83] observations regarding reliability of scores, we also suggest that the lower reliability in subscale scores observed for the VETERAN samples also contribute to the group differences we found in subscale intercepts.

An important question concerns whether observed group differences in item functioning reflect variation in "auxiliary" HRQOL factors or in unrelated "nuisance" factors [9-11]. Given that we have analyzed existing subscales rather than individual items for each instrument, it seems most plausible that group differences reflect "auxiliary" secondary dimensions of HRQOL that are being measured by multiple interrelated subscales and not idiosyncrasies of specific item wording. However, future work is needed to establish this conclusion more definitively (see, e.g., [86].

Our study has several limitations. First, this study was restricted to males between the ages of 28 and 85 living in the United States. This restriction limits the generalizability of our findings to other CKD populations, including females. In addition, the exclusion criteria for the DOPPS and VETERAN data may have been different due to samples from different studies. Furthermore, data were collected from Veterans via telephone interview while data from non-Veterans were collected with a written questionnaire. It is possible that our findings are confounded by these different modes of data collection. Although there is no consensus in the literature related to comparability of data collected via written questionnaires and telephone interviews, several studies have demonstrated that mode of data collection has little to no effect on findings [87-89]. In addition, we did not control for demographic variables such as educational level and household income, which may have confounded the comparison between the VETERANS and DOPPS samples. Furthermore, variables not measured in this study, such as resilience and optimism, may also have contributed to the differences we found between Veterans and non-Veterans in how they interpreted the KDQOL-SF. However, despite not controlling for confounding variables, the generic HRQOL (SF-36) measure maintained cross-group generalizability between the DOPPS and VETERANS samples. Future research is needed using large, randomly selected samples in order to increase generalizability and best address the issue of confounders in the evaluation of instrument measurement invariance. In addition, we only considered subscale scores and did not analyze item-level data. Although SF-36 items have been widely validated, future analyses of KDCS items are needed in order to refine the KDCS to contain only items that produce measurement equivalence within subscales across different cultural groups. Finally, confirmatory factor analysis was the only method used to test for measurement equivalence in this study. There are a variety of statistical methods available to evaluate measurement invariance, such as item response theory (IRT) that may have yielded different results [90]. More research is needed to clarify the impact of different statistical methods to assess measurement invariance.

## Conclusions

Veterans make up a unique cultural group with life experiences in the military that may influence how they interpret HRQOL measures. These potential cultural differences must be considered when comparing perceived HRQOL ratings of Veterans to non-Veterans. Measurement invariance is an essential condition for valid comparisons of HRQOL rated by different cultural groups. Our data supported measurement invariance across Veterans and non-Veterans using the KDQOL-SF. However, structural invariance, a desirable but not necessary precondition for meaningful group comparisons, was not demonstrated, particularly with the KDCS, the disease-specific HRQOL component of the KDQOL-SF. Further evaluation of measurement invariance of the KDQOL-SF between Veterans and non-Veterans is needed using large, randomly selected samples before comparisons between Veterans and non-Veterans using the KDQOL-SF can be done reliably

## Abbreviations

CKD: Chronic Kidney Disease; DOPPS: Dialysis Outcomes and Practice Patterns Study; HRQOL: Health-Related Quality of Life; KDCS: Kidney Disease Component Summary; KDQOL-SF: Kidney Disease Quality of Life - Short Form.

## Competing interests

The authors declare that they have no competing interests.

## Authors' contributions

KLS-Conceptualized paper, contributed to data analyses, and wrote significant sections of manuscript, FBB-Performed data analyses and wrote significant portions of paper including Results section, DJR-Provided statistical consultation for primary study in which data for this paper was derived. Reviewed and commented on drafts of paper, KTS-Investigator for Veteran's study in which data for this study was derived. Reviewed and commented on drafts of paper.

DMH-Senior author and Principal Investigator for the Veteran's study in which these data were derived. Reviewed and commented on drafts of paper.

All authors have read and approved the manuscript.
